# Autism in Viet Nam: A systematic scoping review

**DOI:** 10.1177/13623613261425838

**Published:** 2026-03-08

**Authors:** Leoni Boyle, Prithvi Perepa, Kerry Thalia, Laura Crane

**Affiliations:** 1 University of Birmingham, UK; 2 University of Oxford, UK

**Keywords:** autism, community involvement, evidence synthesis, quality appraisal, reduced inequalities, Viet Nam

## Abstract

Autism research has predominantly focused on Western contexts, with limited studies in Vietnamese cultural settings. Through conducting a systematic scoping review, we aimed to map (a) the landscape of autism research in Vietnamese cultural contexts, (b) the quality of the research, and (c) the extent of autism community involvement in the research. A total of 137 studies met our inclusion criteria. A growing body of literature pertained to autism in Vietnamese cultural contexts, largely conducted in Viet Nam (87%). Much of the literature focused on Services and Supports (39%), as well as Interventions (20%). Key themes identified from the research were the centrality of family, the importance of school and education, and identifying a cause of autism. Quality appraisals of the studies – using the Mixed Methods Appraisal Tool, a Westernised tool – indicated that the studies were largely of low quality. There was limited autism community involvement in the research, with studies often lacking an explicit description of the nature of community involvement. Priority areas for future research include better understanding how rigour is understood in a Vietnamese research context, improving the clarity of data reporting and actively involving the Vietnamese autism community in the research process.

**Lay abstract:**

Autism research has mostly focused on Western contexts, with few studies in Vietnamese cultural contexts. In this study, we reviewed all the research we could find on autism in Vietnamese cultural contexts, to map out what this research ‘looks like’. We found 137 studies on autism in Vietnamese cultural contexts, and most of this research was conducted in Viet Nam. The studies were often focused in the areas of Services and Supports as well as Interventions. Looking for common themes in the research, we found that studies emphasised the importance of family, the importance of school and education, and the need to find the causes of autism. We used the Mixed Methods Appraisal Tool, a Westernised tool, to evaluate the quality of the research, and we found that a lot of the research was rated as ‘low quality’. There were few examples of clear autism community involvement in the research. Key areas for the field to focus on in the future include reflecting on how the quality of the research is evaluated in Global South countries such as Viet Nam, and how best to include the autism community in the research process.

Autism research has been dominated by literature from the Anglosphere and other predominantly Western high-income countries (De Leeuw et al., 2020; Dyches et al., 2004). In autism prevalence studies, for example, fewer studies are conducted in the Global South (Salari et al., 2022; Zeidan et al., 2022) or with Black and ethnic minority populations (Zamora et al., 2016). In relation to autism research with Asian populations, much of the literature represents South Asian and East Asian populations (Qiu et al., 2020; Zeidan et al., 2022) and often homogenises the ‘Asian’ experience. Within the limited research on autism in Southeast Asia, there is an ever-smaller subset of literature pertaining to Viet Nam (Ha et al., 2014; V. M. Hoang et al., 2019; Ilias et al., 2018). Cultural context can influence both conceptualisations of autism and the nature of support for those within the autism community (Ravindran & Myers, 2012). Thus, it is vital to understand research concerning the autism community in Vietnamese cultural contexts.

## The Vietnamese context

In this article, Viet Nam is spelled in accordance with the United Nations M49 Standard, though it should be noted that ‘Vietnam’ is used where it forms part of an organisation’s official name. Viet Nam is a country in Southeast Asia, neighbouring China, Cambodia and Laos, and with a population of approximately 100,990,000 (World Bank, 2024a). Linguistically, Viet Nam is distinctive from its neighbours, with the national language of Vietnamese (Phan Le, 2024). The two largest registered religions are Buddhism (13.3%) and Catholicism (6.6%), though estimates suggest that 90% of the Vietnamese population adhere to some form of religious practice, with most following Vietnamese folk religion and Confucian values of life (Embassy of Vietnam, n.d.; U.S. Department of State, 2024). Officially named the Socialist Republic of Viet Nam, Viet Nam is a one-party state, whereby public infrastructure including schools and hospitals are largely state run (A. V. Le, Han, et al., 2022; Quan & Taylor-Robinson, 2023). Most recent data shows that Viet Nam spends 0.42% of their Gross Domestic Product on Research and Development, ranking them 74th in the world (World Bank, 2024b).

A targeted inquiry into autism research in Vietnamese cultural contexts is lacking, but much needed. We define Vietnamese culture as ‘the set of distinctive spiritual, material, intellectual and emotional features of a society or a social group encompassing, in addition to art and literature, lifestyles, ways of living together, value systems, traditions and beliefs’ (United Nations Educational, Scientific and Cultural Organization [UNESCO], 2002, p. 1). The exploration of Vietnamese cultural contexts allows for Vietnamese diasporas outside of Viet Nam to be accounted for, as cultural legacies from one’s native culture can still shape the perceptions and attitudes of migrants (Bhugra & Becker, 2005; Buriel & De Ment, 1997). In addition, immigrant populations accessing autism support in locations such as North America or Australia have found difficulty in accessing support services, due to linguistic and/or cultural differences (Imanpour et al., 2025; Lim et al., 2021; Luelmo et al., 2020; Smith, Aulich, et al., 2023). It is therefore important for a review on autism within Vietnamese cultural contexts to include culturally Vietnamese populations outside of Viet Nam.

To understand how autism is conceptualised in Viet Nam, it is important to first recognise disability infrastructure in Viet Nam, which can be highlighted in recognition of the first large-scale study of the Vietnamese disabled population, conducted in 2016 and 2017 (Alexander et al., 2024; General Statistics Office [GSO], 2018). Autism as a singular diagnosis is not legislatively recognised as a disability in Viet Nam, though can be recognised when classified as an intellectual disability (ID) or ‘other forms of disability’ (K. K. Hoang, 2025; Truong, 2022). As such, individuals with an autism diagnosis alone might not be able to access support services as they are unable to use disability legislation to uphold their rights (including those relating to health care and education), unless the needs of autistic individuals are understood as a form of ID (Ha et al., 2014; Phan et al., 2020). In addition, an autism diagnosis even within a broader diagnosis of ID was not possible until the late 1990s in Viet Nam, suggesting that autism is a relatively recent phenomenon to be recognised in Viet Nam, often with ambiguity and inconsistency in diagnosis (Ha et al., 2017).

Receiving a diagnosis of autism has been found to often be distressing for the autistic individual as well as their family, due to the stigmatisation of autism in Viet Nam and the resulting social and economic consequences of receiving an autism diagnosis (Boyle & Bailey, 2022; Ha et al., 2014; A. P. Nguyen & Nguyen, 2023; Phan et al., 2020; Yến-Khanh, 2022). Stigmatisation occurs at a linguistic and cultural level, such as the direct Vietnamese translation of autism (‘bệnh tự kỷ’) as ‘autism sickness’, perhaps reflecting that autism is diagnostically recognised by the World Health Organization within the International Classification of Diseases, as well as through the understanding that autism is caused by ancestral sins, karmic demerit and other Vietnamese folk lore narratives (D’Antonio & Shin, 2009; Ha et al., 2014). Generally, global understandings of autism recognise the aetiology of autism to be complex, with interactive genetic and environmental factors likely contributing to markers of autism (Wang et al., 2025). In certain cultural contexts, aetiological understandings of autism not only include complex genetic and environmental interactions but also include conceptualisations of autism as being caused by social and spiritual factors, such as karma and punishment from God in Indian contexts (Vats et al., 2024) and witchcraft in Kenyan contexts (Cloete & Obaigwa, 2019). Given the uniqueness of Vietnamese culture and history and the consequential nuances of the experiences of the Vietnamese autism community, it is thus important to understand the landscape of research that pertains to the autism community in this specific context. The Vietnamese autism community is understood in this review as autistic individuals, their families and caregivers, and professionals who directly interact with the autistic individual (e.g. education or medical professionals).

## Gaps in the literature

To the best of our knowledge, there exists no published review of autism in Vietnamese cultural contexts, and as such there is currently no broad understanding of the landscape of autism research in Vietnamese cultural contexts. In establishing the topography of research conducted in Vietnamese cultural contexts, comparisons can be made as to how the research being conducted compares to research that the global autism community prioritises (primarily in the Western world; Pellicano et al., 2014; Roche et al., 2021). Such an endeavour would allow for future research to better serve the Vietnamese autism community. In addition to establishing the landscape of autism research in Vietnamese cultural contexts, it is important to evaluate the quality of research, particularly as the broader, global field of autism research has often found issues with research quality (Bottema-Beutel et al., 2023; Roche et al., 2021; Russell et al., 2019; Warren et al., 2011). Such an investigation could bolster future empirical research. Furthermore, there is growing recognition of the importance of meaningful and active autism community involvement in research (Fletcher-Watson et al., 2019) alongside clear reporting of such information (Tan et al., 2025). Community involvement is thought to allow for research to better meet the needs of the autism community, allow for more authentic research to be conducted and provide a sense of belonging to the autism community within the field of research (Stark et al., 2021). The need to explore autism community involvement in Vietnamese cultural contexts is made more pertinent considering the importance of diversifying the communities represented in autism research (Cameron et al., 2022; Fletcher-Watson et al., 2019; Pellicano et al., 2014).

The current review aims to address the following research questions:

1. What is the landscape of autism research in Vietnamese cultural contexts (both within and outside of Viet Nam)?
2. What is the quality of autism research within Vietnamese cultural contexts?
3. What is the extent of autism community involvement in autism research in Vietnamese cultural contexts?

## Method

The questions considered in this review are purposefully broad in nature given that the focal enquiry pertains to all autism literature in Vietnamese cultural contexts. As this is the first comprehensive review of autism literature in Vietnamese cultural contexts, a scoping review methodology was employed (Munn et al., 2018; Pollock et al., 2024). Methodological elements pertaining to systematic reviews were also included in this review, notably critical appraisal and synthesis of findings (Munn et al., 2018; Pollock et al., 2024). Consequently, this review adheres to guidance pertaining to systematic scoping reviews (Munn et al., 2018; Peters et al., 2015). The review protocol was pre-registered onto the Open Science Framework on 3 June 2024 (Boyle et al., 2024).

### Positionality statement

The research team consists of a diverse collection of researchers, representing a range of academic expertise in relation to autism, including expertise specifically in culture and ethnicity within autism research. The author team also includes individuals with both lived and professional experience within the autism community, and a direct connection to the Vietnamese cultural context (the first author identifies as Vietnamese-British).

### Search strategy

A key term search was conducted across PsycInfo, SCOPUS, Web of Science, PubMed, ProQuest, CINAHL and Ovid. An adapted key term search was then conducted on Vietnam Journals Online. Studies included in the review were then inputted into ResearchRabbit, to search for further publications of relevance. The searches were conducted by the first author on 12 September 2024, and the searches were updated and re-run on 29 August 2025. The search terms were adapted from criteria employed in similar scoping reviews (Bakare et al., 2022; Dao et al., 2022; Patra & Kar, 2021), incorporating Vietnamese terms identified from Vietnamese literature (Yến-Khanh, 2023b).

The following search was employed in the international database searches. For Vietnam Journals Online, only searches #1 and #2 were conducted as the database accounted for Vietnamese context of the literature. The user interface for Vietnam Journals Online did not allow for traditional searches in code, and instead required a manual approach to conduct the search using each of the different combination of search terms.

#1 autis* OR asperger* OR neurodiver* OR ASD OR ASC OR PDD OR ‘tự kỷ’ OR ‘khuyết tật’ OR ‘hội chứng’

#2 (intellectual* OR learning OR development* OR neurodevelopment* OR cognitive or mental) AND (handicap* OR disab* OR difficult* OR impairment* OR deficien* OR incapacit* OR delay OR disorder* OR retard*)

#3 Vietnam* OR ‘Viet Nam’ OR ‘Việt Nam’

#4 (#1 OR #2) AND #3

### Study selection

Studies were first subject to title and abstract review, and progressed to the full-text review process if either the title and/or abstract met the inclusion criteria, or if there was insufficient information provided for an include/exclude decision to be made. If articles met any of the exclusion criteria at any stage, the article was not selected for review. The first author reviewed all articles collated from the literature search, with 20% of the articles at title and abstract review and at full-text review reviewed by one of five independent coders (Landis & Koch, 1977; Nussbaumer-Streit et al., 2023). Inter-rater reliability was calculated with Cohen’s Kappa, achieving *k* = 0.42 and *k* = 0.90, respectively.

#### Inclusion criteria

The following inclusion criteria were used in study selection:

1. Autism as an explicit area of interest.
2. Research carried out with Vietnamese cultural contexts.
3. Research involving data generation (*including grey literature that generated new data; reviews were not included*).
4. Full text of article available.
5. Paper written in English or Vietnamese.

#### Exclusion criteria

The following exclusion criteria were used in study selection:

1. Studies where it was not possible to extract data pertaining to at least one member of the autism community, distinct from the other data within the study.
2. Reviews, editorials and conference proceedings.

#### PRISMA chart

**Figure 1.**
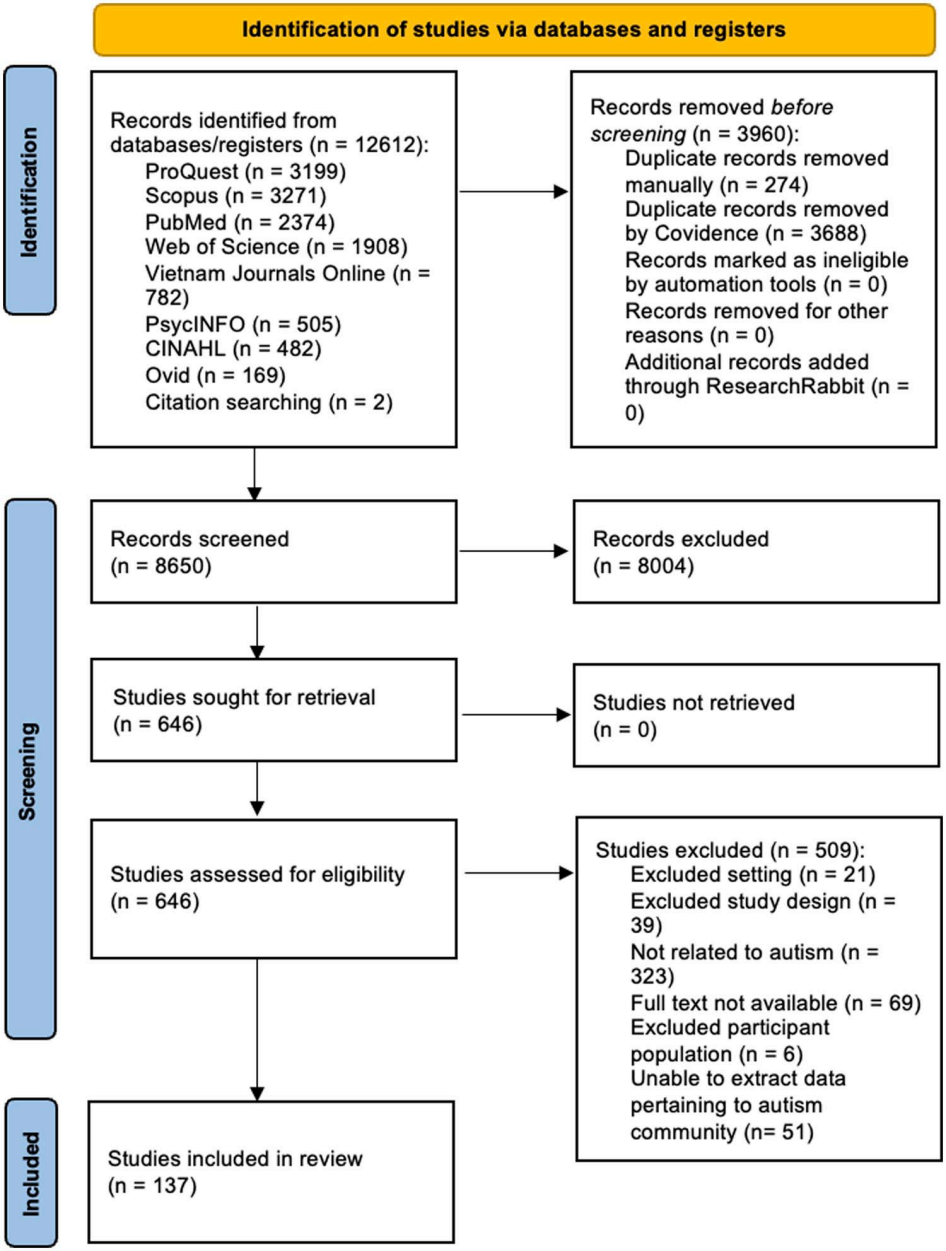
PRISMA flow diagram of literature search strategy.

A total of 12,612 articles were obtained from searches. Following the removal of duplicates, 8650 articles were included at title and abstract review; 646 articles progressed to full-text review; and 137 studies met eligibility criteria and were included in the analysis (Figure 1).

#### Data extraction and data analysis

Data extraction was completed by the first author, using a charting form that recorded key information, including but not limited to: author, publication year, study design, participant position in autism community, thematic area and autism community involvement. Quality appraisal assessment was completed by the first author, using the Mixed Methods Appraisal Tool (MMAT; Hong et al., 2018). Prior to the first author completing data extraction and quality appraisal, the authors collaborated to review the data extraction and MMAT processes using a random selection of studies (*n* = 5) to develop a standardised approach to extraction.

A deductive, thematic area analysis was applied to the studies included in review, assigning each study to one of the seven thematic areas identified by the Interagency Autism Coordinating Committee (IACC, 2016, 2023): Services and Supports, Lifespan, Interventions, Biology, Infrastructure and Surveillance, Screening and Diagnosis, Genetic and Environmental Factors. A further inductive trend analysis was undertaken to provide contextualised insights into the studies included in review. Here, a generic inductive approach was undertaken by the primary author, whereby underlying patterns within the studies included in review were refined to synthesised themes, through iterative reading and annotation of the studies (Liu, 2016; Thomas, 2006). To ensure the rigour and quality of the analysis, the author team collaborated in refining and challenging the findings through collaboration and peer de-briefing (Braun & Clarke, 2006; Nowell et al., 2017).

The second research question, examining the quality of autism research within Vietnamese cultural contexts, was answered using the MMAT. The MMAT is an open-access tool designed in the Global North (Hong et al., 2018), with specific evaluative criteria dependent on the methodology of the study. It is designed as a checklist supported by comments to contextualise the evaluation without a reductive quality score (Oliviera et al., 2021). The MMAT has been found to be reliable and content validated, and is a widely used quality appraisal tool in systematic reviews (Pryjmachuk et al., 2024; Scott et al., 2019; Souto et al., 2015; Wong et al., 2020). As such, the MMAT was considered an appropriate quality appraisal tool for this systematic scoping review.

The third research question concerns the extent of autism community involvement in autism research in Vietnamese cultural contexts. Community involvement in research was assessed based on the explicit reporting of community involvement within the study (collected at data extraction), such as through community involvement statements, description of methods or acknowledgement sections. Community involvement was coded as ‘Yes’, ‘No’ or ‘Unclear’.

## Results

Research question 1, the inquiry into the landscape of autism research in Vietnamese cultural contexts (both within and outside of Viet Nam), was answered through analysis of study characteristics, identification of thematic areas (from the seven IACC themes; IACC, 2016, 2023) and inductive trend analysis.

### Study characteristics

A total of 137 studies were included in the review (Supplementary Materials, Table 1). The first identified article was published in 2008, with 77% (*n* = 106) of the identified studies being published since 2019. Peer-reviewed journal articles made up 81% (*n* = 111) of the articles, whereas 13% (*n* = 18) of articles were published in non-peer-reviewed journals, 4% (*n* = 6) were dissertations and 2% (*n* = 2) were book chapters. Most studies were conducted in Viet Nam (87%, *n* = 120), followed by the United States (7%; *n* = 10) and Australia (3%; *n* = 4), then Finland, the United Kingdom and Norway (each at *n* = 1).

Study design was mixed, though cross-sectional and qualitative studies were the most common (37%, *n* = 50% and 23%, *n* = 32, respectively), often with sample sizes below 200 (Supplementary Materials, Figure 1). The group within the autism community most often represented within Vietnamese cultural contexts were parent/caregivers of autistic children (36%, *n* = 64), followed by autistic individuals themselves (33%, *n* = 59), then educational professionals (20%, *n* = 35). The age of autistic individuals was explicitly reported in 45% (*n* = 61) of the studies included in this review, and in all but one case where age was reported, autistic individuals were within paediatric populations (under the age of 18). There was one study that included a 19-year-old autistic individual (Bá Luyến et al., 2024). In addition, 71% (*n* = 76) of studies with autistic individual and/or parent participants did not explicitly state a formal diagnosis of autism, with those that did referring to both diagnostic manuals and screening tools (Supplementary Materials, Figure 2).

### Thematic areas

**Figure 2.**
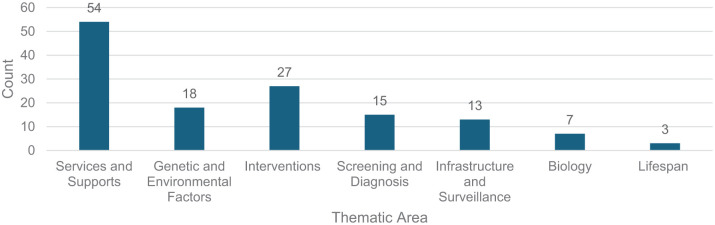
Count of thematic areas based on the IACC categorisation.

The most common thematic area was Services and Supports, followed by Interventions, Genetic and Environmental Factors, Screening and Diagnosis, Infrastructure and Surveillance (Figure 2). The themes of Biology and Lifespan were the least common (Figure 2), and all Lifespan studies were published in 2024–2025.

### Inductive trends

The inductive trend analysis revealed three recurrent themes across the studies, seen as being of particular importance in the Vietnamese cultural context: the centrality of family, the importance of school and education, and identifying a cause of autism.

#### Centrality of family

The experiences of parents and caregivers in raising autistic children was a key area of focus, often exploring their experiences of services and supports, their emotional needs and their quality of life. Across studies, mothers were often identified as the primary caregivers, taking on the role of both participant and data informant (Bogenschutz et al., 2016; Bui, 2017; Jegatheesan, 2009; Pham-The et al., 2022; Poon et al., 2022; Thái & Thứ, 2017; P. N. Thao et al., 2023; N. N. Tran et al., 2016; Vu et al., 2021; Yến-Khanh, 2023a). The centrality of the family was evident in different ways, including being a direct target of inquiry, or implicitly within the findings of the studies. The experiences of the family were often expressed as frustration with accessing the appropriate services for their autistic children. The emotional and financial difficulties experienced in trying to access supports and services, as well as the difficulties experienced in receiving an autism diagnosis and raising an autistic child, were also often explored in depth. A further way in which the centrality of family was illustrated within the research corpus was through the exploration of interventions and supports delivered at the family level, whereby family units and caregivers were either the facilitators or the recipients of interventions and supports (e.g. Inoue et al., 2024; T. L. A. Mai & Chaimongkol, 2022a, 2022b; Thuy et al., 2020). The overriding tone of the studies, reinforcing the centrality of the family, was negative, with an emphasis on the difficulties experienced by family members of an autistic child.

#### Importance of school and education

Schools were often identified as a primary study location (Supplementary Materials, Figure 3). The importance of school and education in the exploration of autism in Vietnamese cultural contexts was illustrated in many ways, such as through studies focusing on teachers and educational professionals as research participants, identifying where and how autistic children are taught, exploring the autism knowledge that teachers and educational professionals have, and perceptions of inclusive education for autistic students. Attitudes towards having autistic students in mainstream classrooms varied, with findings across and within studies showing evidence for both the encouragement of this practice and resistance to mainstream school integration (P. H. Minh, 2020; V. T. T. Nga, 2020; Smith, Rabba, Datta, et al., 2023a; C. V. Tran, Pham, et al., 2020). Despite discrepancies in attitudes towards inclusive education, the importance of education for autistic children more generally was highlighted. In particular, the role of school in educating autistic individuals to be independent, self-sufficient and to develop skills to enable them to contribute to society were emphasised.

#### Identifying a cause of autism

A recurrent theme and narrative in the review was identifying causes of (and factors associated with) autism, and more broadly, autistic behaviours. Genetic and biological causes were a key area of focus, with an emphasis on preventive measures, interventions or ‘treatments’. This finding is aligned with evidence exploring Vietnamese parent perceptions on the aetiology of autism, whereby over 80% of participants endorsed the causal beliefs that child brain structure causes autism, as well as in utero stress/accident (77.3%), and environmental pollution (65.8%; Truong, 2022).

Across the literature that explored the causes of autism, there was repeated, specific identification of the positive association between perinatal dioxin exposure (of 2,3,7,8-tetrachlorodibenzo-p-dioxin) and the development of autistic behaviours. Such studies were explored through the analysis of breast milk dioxin levels and behavioural and cognitive factors in children. There was broad consensus across the studies looking at dioxin exposure and cognitive outcomes, though the exact nature of the relationship differed across studies. For example, significant negative relationships were found between dioxin exposure and neurodevelopment scores (Nishijo et al., 2014; D. T. Thảo et al., 2022), gaze fixation (T. Pham, Nishijo, et al., 2022), motor coordination (N. N. Tran et al., 2016), and mirror neuron system activity (Vu et al., 2021), though significance of dioxin exposure and autistic characteristics varied and effects on girls and boys differed across studies.

### Quality appraisal

Through the application of the MMAT criteria across the studies included in the review, evaluation of studies was largely rated as unclear or not meeting criteria, indicative of poor-quality research (Supplementary Materials, Figure 4). Proportionally, MMAT criteria were most unclear in studies with quantitative descriptive and mixed method methodologies. Of the studies that did not meet the criteria or did not explicitly meet the MMAT criteria, many of the studies were lacking in description of the methodology, data collection, participant characteristics, and justifications of processes and analyses.

### Participatory methods

The third research question examined the extent of autism community involvement in research. In this review, the autism community is defined as autistic individuals, their families and caregivers, and professionals who are directly interacting with the autistic individual (e.g. education or medical professionals). The vast majority of studies did not state any autism community involvement (93%, *n* = 128). A limited number of studies included explicit autism community involvement (3%, *n* = 4) or had unclear autism community involvement (4%, *n* = 5). Within the studies that included autism community involvement, involvement was made explicit through identification of autistic (and non-autistic) researchers (Smith, Rabba, Datta, et al., 2023a), study co-design with the Hanoi Club of Parents of Children with ASD (conducted in Viet Nam; Ha & Whittaker, 2023), through advisory groups of Vietnamese parents of autistic children (conducted in Australia; Smith, Rabba, Dang, et al., 2023a; Smith, Rabba, Datta, et al., 2023b), and through autistic individuals and their family members shaping a PhotoVoice exhibition through which to disseminate the results of the research (conducted in Viet Nam; Ha, 2018). Instances whereby community involvement was unclear had ambiguity in the framing of the wording of the involvement of different members of the autism community, often with no explicit description of their role in the research process but an implication that autism community members were involved. Examples of ambiguity include the autism community involved through the terms ‘support’, ‘collaboration’ and ‘facilitation’, with exact procedures not outlined (Dieu, 2022; Ha et al., 2014; G. T.-H. Le et al., 2024; Poon et al., 2022). In a singular case, the research projects that were reported on in separate articles stated autism community involvement, but the specific involvement in each aspect of the reported studies was unclear (see Ha & Whittaker, 2016).

## Discussion

The number and topic of publications in Vietnamese cultural contexts mirrors the general global trend of research, whereby there has been a steady increase in autism research that focuses heavily on Services and Supports (Office of Autism Research Coordination [OARC], 2012; Roche et al., 2021; Roux et al., 2021; Whyatt & Torres, 2018) and may also reflect broader global trends in striving to understand autism and support autistic people in different cultural contexts (Atherton et al., 2023; De Leeuw et al., 2020; Lee et al., 2025; Papoudi et al., 2021). The current review also found that there was a recent introduction of Lifespan studies to the corpus of autism literature in Vietnamese cultural contexts, which aligns with global autism research where Lifespan studies are increasing (Pellicano et al., 2014). The recent introduction of Lifespan studies might be attributable to autism only being recognised as a diagnosis in Viet Nam in the late 1990s (Ha et al., 2017), thus limiting the ‘Lifespan’ experience that can be recorded at this time in the research. The Lifespan studies in this review all explore vocational skills and career guidance for autistic young people (Bá Luyến et al., 2024; T. Q. H. Nguyễn, 2024; Thị Thu Thủy, 2024), suggesting a focus on skill building and independence through having a job as important concepts within ‘Lifespan’ research in the literature.

The inductive trend analysis yielded three key trends – the centrality of family, the importance of school and education, and identifying a cause of autism. These areas are of particular relevance within a Vietnamese context given Vietnamese cultural values and history. For example, there are strong Vietnamese values rooted in the importance of the family (such as through filial piety, ancestor worship and multigenerational households; H. V. Mai & Le, 2023; T. L. Nguyen & Vu, 2020; Trinh et al., 2017), as well as values emphasising the importance of education (Q. T. N. Nguyen, 2016). The historical context of Viet Nam provides a unique focus on the causes of autism, notably dioxin exposure, whereby tens of millions of gallons of herbicides were used to destroy plantation during the war in Viet Nam, including Agent Orange, much of which was contaminated with 2,3,7,8-tetrachlorodibenzo-*p*-dioxin (TCDD) (Sen, 2022; Stellman & Stellman, 2018). TCDD is carcinogenic to humans, and is a reproductive and developmental toxin (UK Health Security Agency, 2024). The relationship between dioxin exposure and developmental outcomes has been explored in other contexts, namely when dioxin exposure is accidental and a consequence of agricultural or industrial pollution (Marinković et al., 2010; Zheng et al., 2008). However, in the case of Viet Nam, dioxins were intentionally used as chemical warfare (D. T. Le, Pham, & Polachek., 2022; Ngo et al., 2006), and legislative action taken against chemical companies have cited violations of the Geneva Protocol and international law (Le Monde, 2024; Vietnam Association for Victims of Agent Orange/Dioxin v. Dow Chemical Co., 2008). Consequently, the association between dioxin exposure and neurodevelopmental outcomes such as autism might be loaded with the legacies of war, violence and trauma.

There was diversity within the studies in reporting diagnostic information with some citing screening tools and others using diagnostic criteria as evidence for identification of autistic individuals (such as Anh & Hằng, 2018; Hoa et al., 2019; Kế, 2020; K. T. Tran, Le, et al., 2020). Inconsistencies and difficulties in obtaining a formal autism diagnosis in Viet Nam are recognised issues experienced by the Vietnamese autism community (Ha et al., 2014, 2017); a pattern that has also been observed in countries of similar development status, such as Ecuador, India, Bangladesh, and Sri Lanka (Buffle et al., 2025; Hossain et al., 2017).

A further key finding from this review was that most studies did not include autism community involvement. This finding reflects international autism literature, where though there is evidence of an increase in autism community involvement within research, the community calls for greater power in this regard (Cheng et al., 2023; Den Houting et al., 2021; Elsabbagh et al., 2014; Genovesi et al., 2025; Roche et al., 2021; Tan et al., 2025). In this review, 3% of studies included explicit evidence of community involvement in the research. Furthermore, community involvement was unclear in 4% the research, which partially reflects trends found by Tan et al. (2025), whereby a significant minority of studies were unable to be coded for autism community involvement due to ambiguous information. In the current review, ambiguity was often found where studies recognised involvement of members of the autism community, but did not state the extent of their involvement beyond participation.

Community involvement in research is more prevalent in cultural contexts where there is a shift of focus towards disability rights, person-oriented ethics, and trust in institutions and services (Cascio et al., 2020; Chown et al., 2017; Grinker et al., 2012; Pellicano & Stears, 2011). Following the disability rights movement, which calls for disruption to the oppressive nature of research and advocates for more community agency within the research process (Charlton, 1998; Layton et al., 2022), the need for increased and improved autism community involvement in Vietnamese cultural contexts is further politicised in light of colonial historical abuses of power (Hoekstra et al., 2018; Igwe et al., 2022; Mellor, 2022; X. T. Nguyen, 2023). Epistemological concerns regarding power in autism research in Vietnamese cultural contexts should be intersectional, incorporating perspectives from autistic and disability advocacy, as well as from Vietnamese historical perspectives.

### Limitations and future directions

As a consequence of the quantity of literature that was included in the screening process (*n* = 8650), double-coding across the screening and extraction processes was not feasible. The review process was, however, strengthened through 20% of the studies being double-coded at title and abstract and at full-text review, and data extraction and quality appraisal being completed by the primary author following collaboration with co-authors to develop a standardised procedure.

A further limitation of the review was the difficulty in determining the reliability of the peer review process in Vietnamese journals, as all media and press in Viet Nam is regulated (ranking of 174th of 180 in the World Press Freedom Index; Reporters Without Borders, 2024). This issue not only raises potential concerns regarding the accuracy of the reported 81% of studies published in peer-reviewed journals in this review, but might also call into question the reliability of published material from Viet Nam.

A multi-fold limitation in this review was consequence of difficulties in data extraction, whereby studies included in review often lacked explicit recording of key study characteristics and reporting of methodologies. Ambiguity in study characteristics and methodology made it difficult for cross-study comparisons as well as for quality appraisals using the MMAT. Difficulty in applying the MMAT to the corpus of autism literature in Vietnamese cultural contexts also raises questions as to how appropriate the tool is to evaluate studies conducted in this context; a concern reflected in other studies conducted in the Global South that reported poor research quality based on MMAT appraisals (H. N. Le et al., 2025; Singh & Rajak, 2024). Research appraisal tools such as the Aboriginal and Torres Strait Islander quality appraisal tool (Harfield et al., 2020) have been developed to align with particular cultural contexts and is an approach that could be explored in Vietnamese research contexts. Furthermore, evidence suggests that perceptions of knowledge production for scholars trained in Western contexts are more inclined to focus on rigour and journal standards than scholars trained in Viet Nam (C. H. Hoang, 2023; C. H. Hoang & Turner, 2020). Further enquiry into how research quality is conceptualised in Viet Nam is needed, to then develop a more nuanced approach to appraising study quality in Viet Nam.

## Conclusion

The systematic scoping review revealed 137 studies that enabled us to map the body of literature examining autism in Vietnamese cultural contexts, which was broad in content and quality. The review reflected international trends, namely an increase in autism research in recent years, broader issues regarding quality of research and autism community involvement, and Services and Supports being a focal area within the research. The review also revealed trends significant within the Vietnamese cultural context, including the centrality of family, the importance of school and education, and identifying a cause of autism.

The results of the review present a clear message that autism research within Vietnamese cultural contexts is of increasing interest. With this growth of research, there must be a conscious effort to better understand how research quality in Vietnamese cultural settings is conceptualised, to improve the reporting of research methodologies and to increase Vietnamese autism community involvement in research. Autism literature in Vietnamese cultural contexts is in its infancy, though there is opportunity for an innovative and reflexive body of research to be built moving forward.

## Supplemental Material

sj-docx-1-aut-10.1177_13623613261425838 – Supplemental material for Autism in Viet Nam: A systematic scoping review Supplemental material, sj-docx-1-aut-10.1177_13623613261425838 for Autism in Viet Nam: A systematic scoping review by Leoni Boyle, Prithvi Perepa, Kerry Thalia and Laura Crane in Autism

## References

[bibr1-13623613261425838] Alexander J. Hutchinson C. Carey G. (2024). Empowering physically disabled people in Vietnam: A successful microenterprise model. Disabilities, 4(1), 127–143. 10.3390/disabilities4010009

[bibr2-13623613261425838] Anh N. T. Q. Hằng N. T. T. (2018). Phát triển kỹ năng giao tiếp cho trẻ rối loạn phổ tự kỷ: Trường hợp nghiên cứu điển hình. Tạp Chí Khoa Học Và Giáo Dục, 3(47). https://vjol.info.vn/index.php/DHSP-DHH/article/view/38456

[bibr3-13623613261425838] Atherton G. Morimoto Y. Nakashima S. F. Cross L. (2023). Does the study of culture enrich our understanding of autism? A cross-cultural exploration of life on the spectrum in Japan and the West. Journal of Cross-Cultural Psychology, 54(5), 610–634. 10.1177/00220221231169945

[bibr4-13623613261425838] Bakare M. O. Onu J. U. Bello-Mojeed M. A. Okidegbe N. Onu N. N. Munir K. M. (2022). Picture of Autism Spectrum Disorder (ASD) research in West Africa – A scoping review. Research in Autism Spectrum Disorders, 90, 101888. 10.1016/j.rasd.2021.101888

[bibr5-13623613261425838] Bá Luyến N. Thị Thiên H. Thị Minh Hằng V. Nữ Tâm An N . (2024). THỰC TRẠNG HƯỚNG NGHIỆP CHO THANH THIẾU NIÊN RỐI LOẠN PHỔ TỰ KỈ. Journal of Science Educational Science, 69, 157–168. 10.18173/2354-1075.2024-0091a

[bibr6-13623613261425838] Bhugra D. Becker M. A. (2005). Migration, cultural bereavement and cultural identity. World Psychiatry: Official Journal of the World Psychiatric Association (WPA), 4(1), 18–24. 16633496 PMC1414713

[bibr7-13623613261425838] Bogenschutz M. Im H. Liang A. (2016). Ecological model of a good life for people with disabilities in Vietnam. Global Social Welfare, 3(4), 243–254. 10.1007/s40609-016-0068-y

[bibr8-13623613261425838] Bottema-Beutel K. LaPoint S. C. Kim S. Y. Mohiuddin S. Yu Q. McKinnon R. (2023). An evaluation of intervention research for transition-age autistic youth. Autism, 27(4), 890–904. 10.1177/13623613221128761 36189778

[bibr9-13623613261425838] Boyle L. Bailey J. (2022). A case for the reconceptualisation of autism in Vietnam. Southeast Asia Early Childhood Journal, 11(1), 76–105. 10.37134/saecj.vol11.1.6.2022

[bibr10-13623613261425838] Boyle L. Perepa P. Crane L. (2024). Autism in Viet Nam: A systematic scoping review [online]. osf.io/6u9vf 10.1177/13623613261425838 PMC13005892 41795706

[bibr11-13623613261425838] Braun V. Clarke V. (2006). Using thematic analysis in psychology. Qualitative Research in Psychology, 3(2), 77–101. 10.1191/1478088706qp063oa

[bibr12-13623613261425838] Buffle P. Cavadini T. Ortega M. D. L. Armijos C. Soto P. Gentaz E. Crane L. (2025). Journeys towards accessing an autism diagnosis and associated support: A survey of families of autistic children in Ecuador. Autism, 29(3), 596–613. 10.1177/13623613241281029 39340331 PMC11894907

[bibr13-13623613261425838] Bui L. T. (2017). The lived experience of Vietnamese mothers raising a child with autism (Unpublished doctoral dissertation). Wright Institute.

[bibr14-13623613261425838] Buriel R. De Ment T. (1997). Immigration and sociocultural change in Mexican, Chinese, and Vietnamese American families. In Booth A. Crouter A. C. Landale N. S. (Eds.), Immigration and the family: Research and policy on U.S. immigrants (pp. 165–200). Lawrence Erlbaum Associates, Inc.

[bibr15-13623613261425838] Cameron L. A. Borland R. L. Tonge B. J. Gray K. M. (2022). Community participation in adults with autism: A systematic review. Journal of Applied Research in Intellectual Disabilities, 35(2), 421–447. 10.1111/jar.12970 34907624

[bibr16-13623613261425838] Cascio M. A. Weiss J. A. Racine E. & the Autism Research Ethics Task Force. (2020). Person-oriented ethics for autism research: Creating best practices through engagement with autism and autistic communities. Autism, 24(7), 1676–1690. 10.1177/1362361320918763 32551887

[bibr17-13623613261425838] Charlton J. (1998). Nothing about us without us disability oppression and empowerment. University of California Press. 10.1525/california/9780520207950.001.0001

[bibr18-13623613261425838] Cheng Y. Tekola B. Balasubramanian A. Crane L. Leadbitter K. (2023). Neurodiversity and community-led rights-based movements: Barriers and opportunities for global research partnerships. Autism, 27(3), 573–577. 10.1177/13623613231159165 37010065

[bibr19-13623613261425838] Chown N. Robinson J. Beardon L. Downing J. Hughes L. Leatherland J. Fox K. Hickman L. MacGregor D. (2017). Improving research about us, with us: A draft framework for inclusive autism research. Disability & Society, 32(5), 720–734. 10.1080/09687599.2017.1320273

[bibr20-13623613261425838] Cloete L. G. Obaigwa E. O. (2019). Lived experiences of caregivers of children with autism spectrum disorder in Kenya. African Journal of Disability, 8, Article 435. 10.4102/ajod.v8i0.435 PMC6489168 31049306

[bibr21-13623613261425838] D’Antonio E. Shin J. Y. (2009). Families of children with intellectual disabilities in Vietnam: Emerging themes. In International review of research in mental retardation (Vol. 38, pp. 93–123). Elsevier. 10.1016/S0074-7750(08)38004-5

[bibr22-13623613261425838] Dao T. L. To M. M. Nguyen T. D. Hoang V. T. (2022). Mapping COVID-19 related research from Vietnam: A scoping review. Journal of Preventive Medicine and Hygiene, 63(1), E166–E173. 10.15167/2421-4248/jpmh2022.63.1.1720 PMC9121688 35647376

[bibr23-13623613261425838] De Leeuw A. Happé F. Hoekstra R. A . (2020). A conceptual framework for understanding the cultural and contextual factors on autism across the globe. Autism Research, 13(7), 1029–1050. 10.1002/aur.2276 32083402 PMC7614360

[bibr24-13623613261425838] Den Houting J. Higgins J. Isaacs K. Mahony J. Pellicano E . (2021). ‘I’m not just a guinea pig’: Academic and community perceptions of participatory autism research. Autism, 25(1), 148–163. 10.1177/1362361320951696 32854511

[bibr25-13623613261425838] Dieu M. T. (2022). Autism in Vietnam: A three-part study to enhance understanding of Vietnamese parents’ perceptions and etiological beliefs about their children’s autism spectrum disorder [University of Houston]. https://regroup-production.s3.amazonaws.com/documents/ReviewReference/1464749437/TRUONG-DISSERTATION-2022.pdf?X-Amz-Algorithm=AWS4-HMAC-SHA256&X-Amz-Credential=AKIAYSFKCAWYQ4D5IUHG%2F20250909%2Fus-east-1%2Fs3%2Faws4_request&X-Amz-Date=20250909T072139Z&X-Amz-Expires=604800&X-Amz-SignedHeaders=host&X-Amz-Signature=c85c664971d83e2e477e1561d216e4b80f3e6c5519f0fb0912f8b29365b84d89

[bibr26-13623613261425838] Dyches T. T. Wilder L. K. Sudweeks R. R. Obiakor F. E. Algozzine B. (2004). Multicultural issues in autism. Journal of Autism and Developmental Disorders, 34, 211–222. 15162939 10.1023/b:jadd.0000022611.80478.73

[bibr27-13623613261425838] Elsabbagh M. Yusuf A. Prasanna S. Shikako-Thomas K. Ruff C. A. Fehlings M. G. (2014). Community engagement and knowledge translation: Progress and challenge in autism research. Autism, 18(7), 771–781. 10.1177/1362361314546561 25128332

[bibr28-13623613261425838] Embassy of Vietnam. (n.d.). Beliefs and religions. Vietnam Embassy in the United States. https://web.archive.org/web/20100521040905/http://www.vietnamembassy-usa.org/learn_about_vietnam/culture/beliefs_and_religions/

[bibr29-13623613261425838] Fletcher-Watson S. Adams J. Brook K. Charman T. Crane L. Cusack J. Leekam S. Milton D. Parr J. R. Pellicano E. (2019). Making the future together: Shaping autism research through meaningful participation. Autism, 23(4), 943–953. 10.1177/1362361318786721 30095277 PMC6512245

[bibr30-13623613261425838] General Statistics Office. (2018). The national survey on people with disabilities 2016 (VDS2016): Final report. https://www.gso.gov.vn/wp-content/uploads/2019/04/Baocao-nguoikhuyet-tat.pdf

[bibr31-13623613261425838] Genovesi E. Grant S. Kifle T. H. Li J. Shand A. J. Hoekstra R. A. (2025). Community involvement in PhD students’ autism research projects: Challenges and opportunities. Autism, 29(1), 3–7. 10.1177/13623613241300683 39600294

[bibr32-13623613261425838] Grinker R. R. Chambers N. Njongwe N. Lagman A. E. Guthrie W. Stronach S. Richard B. O. Kauchali S. Killian B. Chhagan M. Yucel F. Kudumu M. Barker-Cummings C. Grether J. Wetherby A. M. (2012). ‘Communities’ in community engagement: Lessons learned from autism research in South Korea and South Africa. Autism Research, 5(3), 201–210. 10.1002/aur.1229 22566396 PMC3552431

[bibr33-13623613261425838] Ha V. S. (2018). Case 6.4 Could we protect confidentiality, and for whom? Story from a PhotoVoice project with teenagers living with Autism Spectrum Disorder in Hanoi, Vietnam. In Banks S. Brydon-Miller M. (Eds.), Ethics in participatory research for health and social well-being. Routledge. 10.4324/9781315106847

[bibr34-13623613261425838] Ha V. S. Whittaker A. (2016). ‘Closer to my world’: Children with autism spectrum disorder tell their stories through photovoice. Global Public Health, 11(5–6), 546–563. 10.1080/17441692.2016.1165721 27073986

[bibr35-13623613261425838] Ha V. S. Whittaker A. (2023). ‘Pray to all four directions’: A qualitative study of syncretic care seeking by Vietnamese families for their children with autism spectrum disorder. Disability and Rehabilitation, 45(4), 684–695. 10.1080/09638288.2022.2040613 35234089

[bibr36-13623613261425838] Ha V. S. Whittaker A. Rodger S. (2017). Assessment and diagnosis of autism spectrum disorder in Hanoi, Vietnam. Journal of Child and Family Studies, 26(5), 1334–1344. 10.1007/s10826-017-0655-2

[bibr37-13623613261425838] Ha V. S. Whittaker A. Whittaker M. Rodger S. (2014). Living with autism spectrum disorder in Hanoi, Vietnam. Social Science & Medicine, 120, 278–285. 10.1016/j.socscimed.2014.09.038 25262315

[bibr38-13623613261425838] Harfield S. Pearson O. Morey K. Kite E. Canuto K. Glover K. Gomersall J. S. Carter D. Davy C. Aromataris E. Braunack-Mayer A. (2020). Assessing the quality of health research from an Indigenous perspective: The Aboriginal and Torres Strait Islander quality appraisal tool. BMC Medical Research Methodology, 20(1), Article 79. 10.1186/s12874-020-00959-3 PMC7147059 32276606

[bibr39-13623613261425838] Hoa H. T. Hien T. T. Giang L. T. H. Minh T. H. Mai T. H. Nghi T. N. Hà V. S. (2019). Mô hình sàng lọc rối loạn phát triển và tự kỷ trực tuyến dành cho trẻ từ 9 đến 48 tháng tuổi. Tạp Chí Y Tế Công Cộng, 45. https://vjol.info.vn/index.php/TTCC/article/view/40220

[bibr40-13623613261425838] Hoang C. H. (2023). Glocal production of knowledge: Exploring Vietnamese scholars’ perception of ‘good’ research. Compare: A Journal of Comparative and International Education, 53(1), 123–141. 10.1080/03057925.2021.1884046

[bibr41-13623613261425838] Hoang C. H. Turner M. (2020). Framing Vietnamese scholars’ negotiation of knowledge production: A positioning perspective. Comparative Education, 56(4), 565–582. 10.1080/03050068.2020.1771871

[bibr42-13623613261425838] Hoang K. K. (2025). Policy and legal reform for autism spectrum disorder patients in Vietnam. Journal of Infrastructure, Policy and Development, 9(1), 6521. 10.24294/jipd6521

[bibr43-13623613261425838] Hoang V. M. Le T. V. Chu T. T. Q. Le B. N. Duong M. D. Thanh N. M. Tac Pham V. Minas H. Bui T. T. H. (2019). Prevalence of autism spectrum disorders and their relation to selected socio-demographic factors among children aged 18–30 months in northern Vietnam, 2017. International Journal of Mental Health Systems, 13(1), 29. 10.1186/s13033-019-0285-8 31168317 PMC6487529

[bibr44-13623613261425838] Hoekstra R. A. Girma F. Tekola B. Yenus Z. (2018). Nothing about us without us: The importance of local collaboration and engagement in the global study of autism. BJPsych International, 15(2), 40–43. 10.1192/bji.2017.26 29953134 PMC6020913

[bibr45-13623613261425838] Hong Q. N. Fàbregues S. Bartlett G. Boardman F. Cargo M. Dagenais P. Gagnon M.-P. Griffiths F. Nicolau B. O’Cathain A. Rousseau M.-C. Vedel I. Pluye P. (2018). The Mixed Methods Appraisal Tool (MMAT) version 2018 for information professionals and researchers. Education for Information, 34(4), 285–291. 10.3233/EFI-180221

[bibr46-13623613261425838] Hossain M. D. Ahmed H. U. Jalal Uddin M. M. Chowdhury W. A. Iqbal M. S. Kabir R. I. Chowdhury I. A. Aftab A. Datta P. G. Rabbani G. Hossain S. W. Sarker M. (2017). Autism Spectrum disorders (ASD) in South Asia: A systematic review. BMC Psychiatry, 17(1), Article 281. 10.1186/s12888-017-1440-x PMC5563911 28826398

[bibr47-13623613261425838] Igwe P. A. Madichie N. O. Rugara D. G. (2022). Decolonising research approaches towards non-extractive research. Qualitative Market Research: An International Journal, 25(4), 453–468. 10.1108/QMR-11-2021-0135

[bibr48-13623613261425838] Ilias K. Cornish K. Kummar A. S. Park M. S.-A. Golden K. J. (2018). Parenting stress and resilience in parents of children with Autism Spectrum Disorder (ASD) in Southeast Asia: A systematic review. Frontiers in Psychology, 9, Article 280. 10.3389/fpsyg.2018.00280 PMC5900388 29686632

[bibr49-13623613261425838] Imanpour S. McGehee A. McMaughan D. J. (2025). ‘As immigrants we are all lost in our autism journey’: Experiences of raising children with autism, barriers to equal access, and facilitators to accessing autism services among immigrant fathers. Research in Autism, 124, Article 202588. 10.1016/j.reia.2025.202588

[bibr50-13623613261425838] Inoue M. Yamaguchi H. Nakatani K. Nishimoto A. Namiki K. Kuroda S. Tran T. V. H. Dinh N. T. T. (2024). Effectiveness of online parent training for Vietnamese parents of children with autism spectrum disorders. Yonago Acta Medica, 67(3), 213–224. 10.33160/yam.2024.08.008 39176193 PMC11335923

[bibr51-13623613261425838] Interagency Autism Coordinating Committee. (2016). Interagency Autism Coordinating Committee strategic plan for autism spectrum disorder. https://iacc.hhs.gov/publications/strategic-plan/2023/

[bibr52-13623613261425838] Interagency Autism Coordinating Committee. (2023, September). 2021-2023 /ACC strategic plan for autism research, services, and policy. https://iacc.hhs.gov/publications/strategic-plan/2023/

[bibr53-13623613261425838] Jegatheesan B. (2009). Cross-cultural issues in parent-professional interactions: A qualitative study of perceptions of Asian American mothers of children with developmental disabilities. Research and Practice for Persons with Severe Disabilities, 34(3–4), 123–136. 10.2511/rpsd.34.3-4.123

[bibr54-13623613261425838] Kế ThS. P. N. T . (2020). THỰC TRẠNG VÀ CÁC YẾU TỐ ẢNH HƯỞNG ĐẾN GIÁO DỤC KỸ NĂNG VẬN ĐỘNG CỦA TRẺ TỰ KỶ TẠI THÀNH PHỐ ĐÀ NẴNG. Tạp Chí Khoa Học và Đào Tạo Thể Thao, 14. https://vjol.info.vn/index.php/tdtt/article/view/54968

[bibr55-13623613261425838] Landis J. R. Koch G. G. (1977). The measurement of observer agreement for categorical data. Biometrics, 33(1), 159. 10.2307/2529310 843571

[bibr56-13623613261425838] Layton N. Bould E. Buchanan R. Bredin J. Callaway L. (2022). Inclusive research in health, rehabilitation and assistive technology: Beyond the binary of the ‘researcher’ and the ‘researched’. Social Sciences, 11(6), 233. 10.3390/socsci11060233

[bibr57-13623613261425838] Le A. V. Han P. Khaing M. M. Farrar O. (2022). An emerging dragon: Vietnamese education after Resolution 29. In Reimers F. M. Amaechi U. Banerji A. Wang M. (Eds.), Education to build back better: What can we learn from education reform for a postpandemic world (pp. 99–123). Springer. 10.1007/978-3-030-93951-9_5

[bibr58-13623613261425838] Le D. T. Pham T. M. Polachek S. (2022). The long-term health impact of Agent Orange: Evidence from the Vietnam War. World Development, 155, 105813. 10.1016/j.worlddev.2022.105813

[bibr59-13623613261425838] Le G. T.-H. Rillotta F. Robinson S. (2024). Education and healthcare services for children and young people with intellectual disability in Vietnam: An ecological systems analysis. Disability and Rehabilitation, 47, 2084–2096. 10.1080/09638288.2024.2390664 39145766

[bibr60-13623613261425838] Le H. N. Sofija E. Harris N. Noviasty R. Nguyen T. Phung H. (2025). What strategies are effective to support food security in slow-onset disasters? A mixed-method systematic review of the literature. Food and Energy Security, 14(2), Article e70065. 10.1002/fes3.70065

[bibr61-13623613261425838] Lee J. D. Kang V. Y. Terol A. K. Joo S. (2025). Examining the efficacy of culturally responsive interventions for autistic children and their families: A meta-analysis. Journal of Autism and Developmental Disorders, 55(2), 706–726. 10.1007/s10803-023-06212-2 38246962 PMC11260274

[bibr62-13623613261425838] Le Monde AFP . (2024, August 22). French court dismisses appeal in Agent Orange case. Le Monde. https://www.lemonde.fr/en/police-and-justice/article/2024/08/22/french-court-dismisses-appeal-in-agent-orange-case_6720003_105.html

[bibr63-13623613261425838] Lim N. O’Reilly M. Sigafoos J. Lancioni G. E. Sanchez N. J. (2021). A review of barriers experienced by immigrant parents of children with autism when accessing services. Review Journal of Autism and Developmental Disorders, 8, 366–372. 10.1007/s40489-020-00216-9

[bibr64-13623613261425838] Liu L. (2016). Using generic inductive approach in qualitative educational research: A case study analysis. Journal of Education and Learning, 5(2), 129. 10.5539/jel.v5n2p129

[bibr65-13623613261425838] Luelmo P. Sandoval Y. Kasari C. (2020). Undocumented Mexican mothers of children with autism: Navigating the health care and educational service systems. International Journal of Developmental Disabilities, 68(4), 567–577. 10.1080/20473869.2020.1850159 35937177 PMC9351561

[bibr66-13623613261425838] Mai H. V. Le H. V. (2023). The interdependence of happiness and filial piety within the family: A study in Vietnam. Health Psychology Report, 12(2), 124–132. 10.5114/hpr/172091 38628278 PMC11016942

[bibr67-13623613261425838] Mai T. L. A. Chaimongkol N. (2022a). Effectiveness of a family management intervention program among families of children with autism: A randomized controlled trial. PRIJNR [Internet]. https://he02.tci-thaijo.org/index.php/PRIJNR/article/view/255275

[bibr68-13623613261425838] Mai T. L. A. Chaimongkol N. (2022b). Testing the feasibility of a nursing intervention focusing on family management for caregivers of children with autism. Journal of Health Science and Medical Research, 40. 10.31584/jhsmr.2021830

[bibr69-13623613261425838] Marinković N. Pašalić D. Ferenčak G. Gršković B. Rukavina A. (2010). Dioxins and human toxicity. Archives of Industrial Hygiene and Toxicology, 61(4), 445–453. 10.2478/10004-1254-61-2010-2024 21183436

[bibr70-13623613261425838] Mellor K. (2022). Developing a decolonial gaze: Articulating research/er positionality and relationship to colonial power. Access: Critical Explorations of Equity in Higher Education, 10(1), 26–41. https://novaojs.newcastle.edu.au/ceehe/index.php/iswp/article/view/184

[bibr71-13623613261425838] Minh P. H. (2020). THỰC TRẠNG CÔNG TÁC GIÁO DỤC HÒA NHẬP CHO TRẺ TỰ KỶ Ở CÁC TRƯỜNG MẦM NON TẠI THÀNH PHỐ HỒ CHÍ MINH NHÌN TỪ GÓC ĐỘ CÁC NHÀ QUẢN LÝ CẤP CƠ SỞ. Tạp Chí Khoa Học - Đại Học Văn Lang, 24. https://vjol.info.vn/index.php/tckhvl/article/view/59565

[bibr72-13623613261425838] Munn Z. Peters M. D. J. Stern C. Tufanaru C. McArthur A. Aromataris E. (2018). Systematic review or scoping review? Guidance for authors when choosing between a systematic or scoping review approach. BMC Medical Research Methodology, 18(1), Article 143. 10.1186/s12874-018-0611-x PMC6245623 30453902

[bibr73-13623613261425838] Nga V. T. T. (2020). Need of supporting children with autism and parents in primary schools in Hano. Journal of Science Educational Science, 65(7), 105–113. 10.18173/2354-1075.2020-0082

[bibr74-13623613261425838] Ngo A. D. Taylor R. Roberts C. L. Nguyen T. V. (2006). Association between Agent Orange and birth defects: Systematic review and meta-analysis. International Journal of Epidemiology, 35(5), 1220–1230. 10.1093/ije/dyl038 16543362

[bibr75-13623613261425838] Nguyen A. P. Nguyen L. T. T. (2023). Social work services for autism children in Vietnam: Status quo and challenges. Anthropological Researches and Studies, 13(1), 274–287. 10.26758/13.1.19

[bibr76-13623613261425838] Nguyen Q. T. N. (2016). The Vietnamese values system: A blend of oriental, Western and socialist values. International Education Studies, 9(12), 32. 10.5539/ies.v9n12p32

[bibr77-13623613261425838] Nguyen T. L. Vu H. V. (2020). Ancestor worshiping beliefs in the beliefs and religion life of Vietnamese people: Nature, values, and changes of it in the current period [Preprint]. Preprints. 10.20944/preprints202008.0491.v1

[bibr78-13623613261425838] Nguyễn T. Q. H. (2024). GIÁO DỤC HÀNH VI CHO THIẾU NIÊN RỐI LOẠN PHỔ TỰ KỈ TRONG HOẠT ĐỘNG HƯỚNG NGHIỆP TIẾP CẬN THEO CÁC NGUYÊN TẮC CỦA THIẾT KẾ HỌC TẬP PHỔ DỤNG. Journal of Science Educational Science, 69, 139–147. 10.18173/2354-1075.2024-0089a

[bibr79-13623613261425838] Nguyen X. T. (2023). 10. Decolonial disability studies. In Mills M. Sanchez R. (Eds.), Crip authorship (pp. 108–120). New York University Press. 10.18574/nyu/9781479819386.003.0013

[bibr80-13623613261425838] Nishijo M. Pham T. T. Nguyen A. T. N. Tran N. N. Nakagawa H. Hoang L. V. Tran A. H. Morikawa Y. Ho M. D. Kido T. Nguyen M. N. Nguyen H. M. Nishijo H. (2014). 2,3,7,8-Tetrachlorodibenzo-p-dioxin in breast milk increases autistic traits of 3-year-old children in Vietnam. Molecular Psychiatry, 19(11), 1220–1226. 10.1038/mp.2014.18 24637425

[bibr81-13623613261425838] Nowell L. S. Norris J. M. White D. E. Moules N. J. (2017). Thematic analysis: Striving to meet the trustworthiness criteria. International Journal of Qualitative Methods, 16(1), 1609406917733847. 10.1177/1609406917733847

[bibr82-13623613261425838] Nussbaumer-Streit B. Sommer I. Hamel C. Devane D. Noel-Storr A. Puljak L. Trivella M. Gartlehner G. (2023). Rapid reviews methods series: Guidance on team considerations, study selection, data extraction and risk of bias assessment. BMJ Evidence-Based Medicine, 28(6), 418–423. 10.1136/bmjebm-2022-112185 PMC10715469 37076266

[bibr83-13623613261425838] Office of Autism Research Coordination. (2012). National Institute of Mental Health and Thomson Reuters, Inc. on behalf of the Interagency Autism Coordinating Committee (IACC). IACC/OARC autism spectrum disorder research publications analysis report: The global landscape of autism research. http://iacc.hhs.gov/publications-analysis/july2012/index.shtml

[bibr84-13623613261425838] Oliveira J. L. C. D. Magalhães A. M. M. D. Matsuda L. M. Santos J. L. G. D. Souto R. Q. Riboldi C. D. O. Ross R. (2021). Mixed methods appraisal tool: Strengthening the methodological rigor of mixed methods research studies in nursing. Texto & Contexto–Enfermagem, 30, Article e20200603. 10.1590/1980-265x-tce-2020-0603

[bibr85-13623613261425838] Papoudi D. Jørgensen C. R. Guldberg K. Meadan H. (2021). Perceptions, experiences, and needs of parents of culturally and linguistically diverse children with autism: A scoping review. Review Journal of Autism and Developmental Disorders, 8(2), 195–212. 10.1007/s40489-020-00210-1

[bibr86-13623613261425838] Patra S. Kar S. K. (2021). Autism spectrum disorder in India: A scoping review. International Review of Psychiatry, 33(1–2), 81–112. 10.1080/09540261.2020.1761136 32602754

[bibr87-13623613261425838] Pellicano E. Dinsmore A. Charman T. (2014). What should autism research focus upon? Community views and priorities from the United Kingdom. Autism, 18(7), 756–770. 10.1177/1362361314529627 24789871 PMC4230972

[bibr88-13623613261425838] Pellicano E. Stears M. (2011). Bridging autism, science and society: Moving toward an ethically informed approach to autism research. Autism Research, 4(4), 271–282. 10.1002/aur.201 21567986

[bibr89-13623613261425838] Peters M. D. J. Godfrey C. M. Khalil H. McInerney P. Parker D. Soares C. B. (2015). Guidance for conducting systematic scoping reviews. International Journal of Evidence-based Healthcare, 13(3), 141–146. 10.1097/XEB.0000000000000050 26134548

[bibr90-13623613261425838] Pham-The T. Nishijo M. Pham T. N. Vu H. T. Tran N. N. Tran A. H. Hoang L. V. Do Q. Nishino Y. Nishijo H. (2022). Perinatal dioxin exposure and Attention Deficit Hyperactivity Disorder (ADHD) symptoms in children living in a dioxin contamination hotspot in Vietnam. Toxics, 10(5), 212. 10.3390/toxics10050212 35622626 PMC9143824

[bibr91-13623613261425838] Pham T. Nishijo M. Pham-The T. Tran N. Vu H. Tran A. Tran T. Nishino Y. Nishijo H. (2022). Effect of perinatal dioxin exposure originating from Agent Orange on gaze behavior in 3-year-old children living in the most dioxin-contaminated areas in Vietnam. Toxics, 10(4), 150. 10.3390/toxics10040150 35448411 PMC9032459

[bibr92-13623613261425838] Pham T. N. H. Phạm A. T. Trần N. H. Lê T. X. Nguyễn T. L. Nguyễn T. H. (2022). Cao chiết Rau đắng biển (Bacopa monnieri) cải thiện hành vi tự kỷ theo cơ chế liên quan đến protein PTEN trên mô hình chuột thực nghiệm gây bởi muối natri valproat. Vietnam Journal of Science and Technology, 64(5B).

[bibr93-13623613261425838] Phan H. Freidman A. Lien N. Q. (2020). Report on reviewing Vietnam’s Law on persons with disabilities in comparison with the convention on the rights of persons with disabilities and international best practices.

[bibr94-13623613261425838] Phan Le H . (2024). Foregrounding Vietnamese language, education, and change in and outside Vietnam. In Phan Le H. Bao D. Windle J. (Eds.), Vietnamese language, education and change in and outside Vietnam (pp. 1–9). Springer Nature Singapore. 10.1007/978-981-99-9093-1_1

[bibr95-13623613261425838] Pollock D. Evans C. Jia R. M. Alexander L. Pieper D. Brandão de Moraes É. Peters M. D. J. Tricco A. C. Khalil H. Godfrey C. M. Saran A. Campbell F. Munn Z. (2024). ‘How-to’: Scoping review? Journal of Clinical Epidemiology, 176, Article 111572. 10.1016/j.jclinepi.2024.111572 39426499

[bibr96-13623613261425838] Poon A. W. C. Cassaniti M. Karan P. Ow R. (2022). Perceived needs and wellbeing of Vietnamese parents caring for children with disability. Children and Youth Services Review, 136, 106433. 10.1016/j.childyouth.2022.106433

[bibr97-13623613261425838] Pryjmachuk S. Kirk S. Fraser C. Evans N. Lane R. Neill L. Camacho E. Bower P. Bee P. McDougall T. (2024). Service design for children and young people with common mental health problems: Literature review, service mapping and collective case study. Health and Social Care Delivery Research, 12(13). 10.3310/DKRT6293 38767587

[bibr98-13623613261425838] Qiu S. Lu Y. Li Y. Shi J. Cui H. Gu Y. Li Y. Zhong W. Zhu X. Liu Y. Cheng Y. Liu Y. Qiao Y. (2020). Prevalence of autism spectrum disorder in Asia: A systematic review and meta-analysis. Psychiatry Research, 284, 112679. 10.1016/j.psychres.2019.112679 31735373

[bibr99-13623613261425838] Quan N. K. Taylor-Robinson A. W. (2023). Vietnam’s evolving healthcare system: Notable successes and significant challenges. Cureus, 15, Article e40414. 10.7759/cureus.40414 PMC10348075 37456482

[bibr100-13623613261425838] Ravindran N. Myers B. J. (2012). Cultural influences on perceptions of health, illness, and disability: A review and focus on autism. Journal of Child and Family Studies, 21(2), 311–319. 10.1007/s10826-011-9477-9

[bibr101-13623613261425838] Reporters Without Borders. (2024). Vietnam. RSF. https://rsf.org/en/country/vietnam

[bibr102-13623613261425838] Roche L. Adams D. Clark M. (2021). Research priorities of the autism community: A systematic review of key stakeholder perspectives. Autism, 25(2), 336–348. 10.1177/1362361320967790 33143455

[bibr103-13623613261425838] Roux A. M. Rast J. E. Garfield T. Shattuck P. Shea L. L. (2021). National autism indicators report: Family perspectives on services and support. A.J. Drexel Autism Institute. 10.17918/FAMILYPERSPECTIVES2021

[bibr104-13623613261425838] Russell G. Mandy W. Elliott D. White R. Pittwood T. Ford T. (2019). Selection bias on intellectual ability in autism research: A cross-sectional review and meta-analysis. Molecular Autism, 10(1), 9. 10.1186/s13229-019-0260-x 30867896 PMC6397505

[bibr105-13623613261425838] Salari N. Rasoulpoor S. Rasoulpoor S. Shohaimi S. Jafarpour S. Abdoli N. Khaledi-Paveh B. Mohammadi M. (2022). The global prevalence of autism spectrum disorder: A comprehensive systematic review and meta-analysis. Italian Journal of Pediatrics, 48(1), 112. 10.1186/s13052-022-01310-w 35804408 PMC9270782

[bibr106-13623613261425838] Scott S. D. Rotter T. Flynn R. Brooks H. M. Bannar-Martin K. H. (2019). Process evaluation of process evaluations in knowledge translation research: A systematic review. Systematic Reviews, 8, Article 266. 10.1186/s13643-019-1161-y PMC6836407 31699136

[bibr107-13623613261425838] Sen A. K. (2022). Addressing the harmful legacy of Agent Orange in Vietnam. https://www.usip.org/publications/2022/01/addressing-harmful-legacy-agent-orange-vietnam

[bibr108-13623613261425838] Singh S. Rajak R. (2024). Barriers in utilization and provisioning of obstetric care services (OCS) in India: A mixed-methods systematic review. BMC Pregnancy and Childbirth, 24(1), Article 16. 10.1186/s12884-023-06189-x PMC10759396 38166775

[bibr109-13623613261425838] Smith J. A. Aulich A. Bent C. A. Constantine C. Franks K. Goonetilleke N. Green C. C. Lee P. Ma E. Said H. Wang R. Wood S. Hudry K. (2023). ‘What is early intervention? I had no idea’: Chinese parents’ experiences of early supports for their autistic children in Australia. Research in Autism Spectrum Disorders, 120, Article 102227. 10.1016/j.rasd.2023.102227

[bibr110-13623613261425838] Smith J. A. Rabba A. S. Dang N. Datta P. Dresens E. Nguyen H. T. T. Nguyen K.-V. Nguyen P. Hall G. Heyworth M. Lawson W. Lilley R. Syeda N. Pellicano E. (2023a). ‘We don’t make trouble’: Vietnamese parents’ experiences of parent-teacher partnerships for their autistic children. Research in Autism Spectrum Disorders, 103, 102142. 10.1016/j.rasd.2023.102142

[bibr111-13623613261425838] Smith J. A. Rabba A. S. Datta P. Dresens E. Wang R. Cong L. Dang N. Hall G. Heyworth M. Lawson W. Lee P. Lilley R. Ma E. Nguyen H. T. T. Nguyen K.-V. Nguyen P. Yeow C. T. Pellicano E. (2023b). ‘It’s really important to be collaborating’: Experiences of participatory research for Chinese and Vietnamese parents of autistic children. Autism & Developmental Language Impairments, 8, 23969415231210482. 10.1177/23969415231210482 PMC10644728 38028582

[bibr112-13623613261425838] Souto R. Q. Khanassov V. Hong Q. N. Bush P. L. Vedel I. Pluye P. (2015). Systematic mixed studies reviews: Updating results on the reliability and efficiency of the Mixed Methods Appraisal Tool. International Journal of Nursing Studies, 52(1), 500–501. 10.1016/j.ijnurstu.2014.08.010 25241931

[bibr113-13623613261425838] Stark E. Ali D. Ayre A. Schneider N. Parveen S. Marais K. Holmes N. Pender R. (2021). Coproduction with autistic adults: Reflections from the authentistic research collective. Autism in Adulthood: Challenges and Management, 3(2), 195–203. 10.1089/aut.2020.0050 PMC8992895 36601467

[bibr114-13623613261425838] Stellman J. M. Stellman S. D. (2018). Agent Orange during the Vietnam War: The lingering issue of its civilian and military health impact. American Journal of Public Health, 108(6), 726–728. 10.2105/AJPH.2018.304426 29741935 PMC5944896

[bibr115-13623613261425838] Tan D. W. Crane L. Haar T. Heyworth M. Poulsen R. Pellicano E. (2025). Reporting community involvement in autism research: Findings from the journal Autism. Autism, 29(2), 490–503. 10.1177/13623613241275263 39239858 PMC11816470

[bibr116-13623613261425838] Thái P. T. H. Thứ H. T. (2017). KHÓ KHĂN TÂM LÝ CỦA NHỮNG BÀ MẸ TRONG VIỆC CHĂM SÓC CON MẮC CHỨNG TỰ KỶ. TẠP CHÍ KHOA HỌC ĐẠI HỌC VĂN HIẾN, 5(4).

[bibr117-13623613261425838] Thảo D. T. Quach Huyen T. Do Thi T. Tran Thi Bich N. Nguyen Hoai T. Nguyen Cong K. (2022). Clarity in social intelligence of secondary school students developing communicative skills for children with autism spectrum disorders learning in inclusive middle schools. Journal of Science Educational Science, 67(2), 71–81. 10.18173/2354-1075.2022-0024

[bibr118-13623613261425838] Thao P. N. Nishijo M. Tai P. T. Nghi T. N. Hoa V. T. Anh T. H. Tien T. V. Nishino Y. Nishijo H. (2023). Impacts of perinatal dioxin exposure on gaze behavior in 2-year-old children in the largest dioxin-contaminated area in Vietnam. Scientific Reports, 13(1), 20679. 10.1038/s41598-023-47893-0 PMC10673870 38001134

[bibr119-13623613261425838] Thị Thu Thủy Đ . (2024). ỨNG DỤNG MÔ HÌNH THIẾT KẾ PHỔ QUÁT (UDL) TRONG GIÁO DỤC HƯỚNG NGHIỆP CHO THANH THIẾU NIÊN RỐI LOẠN PHỔ TỰ KỈ. Journal of Science Educational Science, 69, 148–156. 10.18173/2354-1075.2024-0090a

[bibr120-13623613261425838] Thomas D. R. (2006). A general inductive approach for qualitative data analysis. American Journal of Evaluation, 27(2), 237–246. 10.1177/1098214005283748

[bibr121-13623613261425838] Thuy H. T. Chi N. T. Q. Nga N. T. Ha D. T. Giang N. T. H. Huong N. T. (2020). Developing a community-based autism spectrum disorder management model: Results after 1-year pilot experience. International Journal of Healthcare Management, 14(4), 1181–1189. 10.1080/20479700.2020.1755809

[bibr122-13623613261425838] Tran C. V. Pham M. M. Mai P. T. Le T. T. Nguyen D. T. (2020). Inclusive education for students with autism spectrum disorder in elementary schools in Vietnam: The current situation and solutions. International Electronic Journal of Elementary Education, 12(3), 265–273. https://www.iejee.com/index.php/IEJEE/article/view/1089

[bibr123-13623613261425838] Tran K. T. Le V. S. Bui H. T. P. Do D. H. Ly H. T. T. Nguyen H. T. Dao L. T. M. Nguyen T. H. Vu D. M. Ha L. T. Le H. T. T. Mukhopadhyay A. Nguyen L. T. (2020). Genetic landscape of autism spectrum disorder in Vietnamese children. Scientific Reports, 10(1), 5034. 10.1038/s41598-020-61695-8 32193494 PMC7081304

[bibr124-13623613261425838] Tran N. N. Pham T. T. Ozawa K. Nishijo M. Nguyen A. T. N. Tran T. Q. Hoang L. V. Tran A. H. Phan V. H. A. Nakai A. Nishino Y. Nishijo H. (2016). Impacts of perinatal dioxin exposure on motor coordination and higher cognitive development in Vietnamese preschool children: A five-year follow-up. PLOS ONE, 11(1), Article e0147655. 10.1371/journal.pone.0147655 PMC4732982 26824471

[bibr125-13623613261425838] Trinh Q. T. Kendig H. L. Yiengprugsawan V. (2017). Changes in living arrangements of Vietnamese older adults [Abstract]. Innovation in Aging, 1(Suppl. 1), 378. 10.1093/geroni/igx004.1374

[bibr126-13623613261425838] Truong D. M. (2022). Autism in Vietnam: A three-part study to enhance understanding of Vietnamese parents’ perceptions and etiological beliefs about their children’s autism spectrum disorder [Doctoral dissertation, University of Houston]. University of Houston Institutional Repository.

[bibr127-13623613261425838] UK Health Security Agency. (2024). Dioxins: Toxicological overview. GOV.UK. https://www.gov.uk/government/publications/dioxins-properties-incident-management-and-toxicology/dioxins-toxicological-overview

[bibr128-13623613261425838] United Nations Educational, Scientific and Cultural Organization. (2002). Universal declaration on cultural diversity. https://unesdoc.unesco.org/ark:/48223/pf0000127162

[bibr129-13623613261425838] U.S. Department of State. (2024, June 26). 2023 report on international religious freedom: Vietnam. https://www.state.gov/reports/2023-report-on-international-religious-freedom/vietnam/

[bibr130-13623613261425838] Vats T. Sinha A. K. Arun P. (2024). Exploring the intersections of cultural constructs and parental beliefs: Autism spectrum disorder in Indian families. The Oriental Anthropologist: A Biannual International Journal of the Science of Man, 24, 262–281. 10.1177/0972558X241260830

[bibr131-13623613261425838] *Vietnam Association for Victims of Agent Orange/Dioxin v. Dow Chemical Co.*, 517 F.3d 104 (2d Cir. 2008). https://www.refworld.org/jurisprudence/caselaw/usaca2/2008/en/55023

[bibr132-13623613261425838] Vu H. T. Nishijo M. Pham T. N. Pham-The T. Hoanh L. V. Tran A. H. Tran N. N. Nishino Y. Do Q. Nishijo H. (2021). Effects of perinatal dioxin exposure on mirror neuron activity in 9-year-old children living in a hot spot of dioxin contamination in Vietnam. Neuropsychologia, 161, 108001. 10.1016/j.neuropsychologia.2021.108001 34450135

[bibr133-13623613261425838] Wang M. Zhang X. Zhong L. Zeng L. Li L. Yao P. (2025). Understanding autism: Causes, diagnosis, and advancing therapies. Brain Research Bulletin, 227, Article 111411. 10.1016/j.brainresbull.2025.111411 40449388

[bibr134-13623613261425838] Warren Z. McPheeters M. L. Sathe N. Foss-Feig J. H. Glasser A. Veenstra-VanderWeele J. (2011). A systematic review of early intensive intervention for autism spectrum disorders. Pediatrics, 127(5), e1303–e1311. 10.1542/peds.2011-0426 21464190

[bibr135-13623613261425838] Whyatt C. P. Torres E. B. (2018). Autism research: An objective quantitative review of progress and focus between 1994 and 2015. Frontiers in Psychology, 9, Article 1526. 10.3389/fpsyg.2018.01526 PMC6116169 30190695

[bibr136-13623613261425838] Wong E. Mavondo F. Fisher J. (2020). Patient feedback to improve quality of patient-centred care in public hospitals: A systematic review of the evidence. BMC Health Services Research, 20, Article 530. 10.1186/s12913-020-05383-3 PMC7291559 32527314

[bibr137-13623613261425838] World Bank. (2024a). Population, total for Viet Nam [Data set]. https://data.worldbank.org/indicator/SP.POP.TOTL?locations=VN

[bibr138-13623613261425838] World Bank. (2024b). Research and development expenditure (% of GDP)–Vietnam [Data set]. https://data.worldbank.org/indicator/GB.XPD.RSDV.GD.ZS?locations=VN

[bibr139-13623613261425838] Yến-Khanh N. (2022). The blame game in a child abuse incident in Vietnamese online news media: A framing analysis. Communication and the Public, 7(2), 84–96. 10.1177/20570473221094052

[bibr140-13623613261425838] Yến-Khanh N. (2023a). Medicalized motherhood and normalized autism: A legacy of the eugenic ideology in media stories in authoritarian Vietnam. Journal of Communication Inquiry, 49, 529–546. 10.1177/01968599231176494

[bibr141-13623613261425838] Yến-Khanh N. (2023b). Representation of autism in Vietnamese digital news media: A computational corpus and framing analysis. Communication Research and Practice, 9(2), 142–158. 10.1080/22041451.2023.2167510

[bibr142-13623613261425838] Zamora I. Williams M. E. Higareda M. Wheeler B. Y. Levitt P. (2016). Brief report: Recruitment and retention of minority children for autism research. Journal of Autism and Developmental Disorders, 46(2), 698–703. 10.1007/s10803-015-2603-6 26404703

[bibr143-13623613261425838] Zeidan J. Fombonne E. Scorah J. Ibrahim A. Durkin M. S. Saxena S. Yusuf A. Shih A. Elsabbagh M. (2022). Global prevalence of autism: A systematic review update. Autism Research, 15(5), 778–790. 10.1002/aur.2696 35238171 PMC9310578

[bibr144-13623613261425838] Zheng G. J. Leung A. O. W. Jiao L. P. Wong M. H. (2008). Polychlorinated dibenzo-p-dioxins and dibenzofurans pollution in China: Sources, environmental levels and potential human health impacts. Environment International, 34(7), 1050–1061. 10.1016/j.envint.2008.02.011 18440070

