# Supplementary material for: Autism in Viet Nam: A systematic scoping review
Source: Autism. 2026 Mar 8;30(4):1073–87. doi: 10.1177/13623613261425838 (PMC13005892; doi:10.1177/13623613261425838)
Supplement: sj-docx-1-aut-10.1177_13623613261425838 – Supplemental material for Autism in Viet Nam: A systematic scoping review [file sj-docx-1-aut-10.1177_13623613261425838.docx]

**Supplementary Materials**

**Table 1.**

Studies Included in Systematic Scoping Review.

| **Study** | **Publication Type** | **Country in which the study was conducted** | **Study setting** | **Study design** | **Formal autism diagnosis** | **Thematic area** |
| --- | --- | --- | --- | --- | --- | --- |
| Thảo et al*.* (2022) | Peer Reviewed Journal | Viet Nam | School | Other: Pre- and post- test | Unclear | Interventions |
| Hà (2019) | Not Peer Reviewed Journal | Viet Nam | School | Other: Case study | Unclear | Services and Supports |
| Đinh et al. (2020) | Peer Reviewed Journal | Viet Nam | Other: Commune health centre | Cross sectional study | Unclear | Services and Supports |
| Thương, Do Thi, and Le Thi (2021) | Peer Reviewed Journal | Viet Nam | School; Other: Specialised centres | Cross sectional study | Unclear | Interventions |
| Kế (2020) | Not Peer Reviewed Journal | Viet Nam | School | Cross sectional study | Unclear | Services and Supports |
| Dung (2023) | Not Peer Reviewed Journal | Viet Nam | School | Cross sectional study | Unclear | Interventions |
| Phương and Phi (2019) | Not Peer Reviewed Journal | Viet Nam | School | Cross sectional study | Unclear | Infrastructure and Surveillance |
| Hằng, Hằng, Hà, and Anh (2019) | Peer Reviewed Journal | Viet Nam | School | Cross sectional study | Unclear | Biology |
| Dương (2020) | Not Peer Reviewed Journal | Viet Nam | School | Qualitative research | No | Interventions |
| Minh (2023) | Not Peer Reviewed Journal | Viet Nam | School | Cross sectional study | Unclear | Services and Supports |
| Hòa and Kế (2023) | Not Peer Reviewed Journal | Viet Nam | School; Other: Early intervention centres | Cross sectional study | No | Services and Supports |
| Minh (2020) | Not Peer Reviewed Journal | Viet Nam | School | Other: Descriptive study | Unclear | Services and Supports |
| Hài, Bền, and Tuấn (2022) | Not Peer Reviewed Journal | Viet Nam | School | Cross sectional study | Unclear | Interventions |
| Lan (2012a) | Peer Reviewed Journal | Viet Nam | Unclear | Cross sectional study | Unclear | Services and Supports |
| Hứa et al*.* (2020) | Peer Reviewed Journal | Viet Nam | Hospital | Non-randomised experimental study | No | Interventions |
| Xuân and Ngọc (2024) | Not Peer Reviewed Journal | Viet Nam | School | Cross sectional study | No | Services and Supports |
| Anh and Hằng (2018) | Peer Reviewed Journal | Viet Nam | Other: Home | Other: Case study | Unclear | Interventions |
| Ha and Uyen (2020) | Peer Reviewed Journal | Viet Nam | School | Other: Case study | Unclear | Services and Supports |
| Nga (2012) | Peer Reviewed Journal | Viet Nam | School | Cross sectional study | No | Services and Supports |
| Mai (2012) | Peer Reviewed Journal | Viet Nam | Unclear | Cross sectional study | No | Screening and Diagnosis |
| Huyễn (2019) | Dissertation | Viet Nam | Tertiary care medical centre; Hospital | Qualitative research | Unclear | Services and Supports |
| Nga (2020) | Peer Reviewed Journal | Viet Nam | School | Qualitative research | No | Services and Supports |
| Phu (2022) | Peer Reviewed Journal | Viet Nam | School | Qualitative research | Unclear | Services and Supports |
| Điệp (2008) | Peer Reviewed Journal | Viet Nam | Unclear | Prevalence study | Unclear | Biology |
| Lan (2012b) | Peer Reviewed Journal | Viet Nam | Unclear | Cross sectional study | Unclear | Infrastructure and Surveillance |
| Dung, Anh, Khương, Ngân, and Tuyên (2023) | Not Peer Reviewed Journal | Viet Nam | Hospital | Case control study | Yes | Genetic and Environmental Factors |
| Băng (2023) | Peer Reviewed Journal | Viet Nam | Other | Other: | No | Interventions |
| Điệp (2015) | Peer Reviewed Journal | Viet Nam | Unclear | Cross sectional study | Unclear | Biology |
| Hồng (2024) | Peer Reviewed Journal | Viet Nam | Unclear | Other: | Unclear | Services and Supports |
| Tuyền, Trúc, and Thuộc (2021) | Not Peer Reviewed Journal | Viet Nam | Unclear | Cross sectional study | No | Services and Supports |
| Hoa et al. (2018) | Peer Reviewed Journal | Viet Nam | Unclear; Other: Online | Other: | Unclear | Screening and Diagnosis |
| Anh, Anh, Hoàng, and Giang (2020) | Not Peer Reviewed Journal | Viet Nam | Other: Home | Cross sectional study | No | Services and Supports |
| Phạm and Huỳnh (2017) | Not Peer Reviewed Journal | Viet Nam | Unclear | Qualitative research | Unclear | Services and Supports |
| Công and Điệp (2017) | Peer Reviewed Journal | Viet Nam | School | Non-randomised experimental study | Unclear | Interventions |
| Băng (2024) | Peer Reviewed Journal | Viet Nam | Unclear | Other: | No | Interventions |
| Khánh (2018) | Peer Reviewed Journal | Viet Nam | Other: University | Qualitative research | Unclear | Infrastructure and Surveillance |
| Đạt and Tuệ (2020) | Not Peer Reviewed Journal | Viet Nam | Unclear | Randomised controlled trial | No | Genetic and Environmental Factors |
| Hoa, Huyền, Lan, Huyền, and Luyên (2022) | Peer Reviewed Journal | Viet Nam | Other: Specialised education centres | Cross sectional study | Unclear | Interventions |
| Kế (2022) | Peer Reviewed Journal | Viet Nam | School | Cross sectional study | No | Interventions |
| Thu, Huyen, Ut, Dung, and Thu (2020) | Peer Reviewed Journal | Viet Nam | Unclear | Other: Case study | Unclear | Interventions |
| Hài, Bền, and Tuấn (2021) | Peer Reviewed Journal | Viet Nam | School | Cross sectional study | No | Interventions |
| Hằng et al. (2021) | Peer Reviewed Journal | Viet Nam | Other: Lab | Randomised controlled trial | No | Biology |
| Xuân et al. (2022) | Not Peer Reviewed Journal | Viet Nam | Unclear | Case control study | Unclear | Genetic and Environmental Factors |
| Luong, Yoder, and Canham (2009) | Peer Reviewed Journal | United States | Unclear | Qualitative research | Unclear | Services and Supports |
| Wakamatsu (2022) | Peer Reviewed Journal | Australia | Unclear | Other: Case study | Unclear | Interventions |
| Austin (2011) | Peer Reviewed Journal | Viet Nam | School; Other: Workshop (for credit by Ministry of Education and Training ), and University | Qualitative research | Unclear | Services and Supports |
| Nguyen (2020) | Dissertation | United States | Unclear | Qualitative research | Unclear | Services and Supports |
| Becerra (2013) | Dissertation | United States | Other: California Department of Developmental Services | Case control study | Yes | Genetic and Environmental Factors |
| Bui (2017) | Dissertation | United States | Unclear | Qualitative research | Yes | Services and Supports |
| Bui (2021) | Dissertation | Viet Nam | Hospital | Case control study | Yes | Genetic and Environmental Factors |
| Wiszniewskiet al. (2013) | Peer Reviewed Journal | United States | Hospital | Cross sectional study | Unclear | Genetic and Environmental Factors |
| Van Tran and Weiss (2018) | Peer Reviewed Journal | Viet Nam | Unclear | Qualitative research | Unclear | Services and Supports |
| Thi Vui et al. (2022) | Peer Reviewed Journal | Viet Nam | Unclear | Cross sectional study | Yes | Screening and Diagnosis |
| Than et al. (2023) | Peer Reviewed Journal | Viet Nam | Hospital | Cohort study | Yes | Interventions |
| Thuong, Van Thu, and Van Hieu (2021) | Peer Reviewed Journal | Viet Nam | Tertiary care medical centre; School | Cross sectional study | No | Services and Supports |
| Vu et al. (2021) | Peer Reviewed Journal | Viet Nam | Other | Cohort study | Unclear | Genetic and Environmental Factors |
| Jegatheesan (2009) | Peer Reviewed Journal | United States | Other: Parent Organisations | Qualitative research | Unclear | Services and Supports |
| Bogenschutz, Im, and Liang (2016) | Peer Reviewed Journal | Viet Nam | Non-Governmental Organizations (NGO) | Qualitative research | Unclear | Services and Supports |
| Son, Moring, Igdalsky, and Parish (2018) | Peer Reviewed Journal | United States | Other: Community agency | Qualitative research | Unclear | Services and Supports |
| Phan et al*.* (2022) | Peer Reviewed Journal | Viet Nam | Tertiary care medical centre | Cross sectional study | Unclear | Biology |
| Inoue et al. (2024) | Peer Reviewed Journal | Viet Nam | Other: University | Cohort study | Unclear | Services and Supports |
| Lehti et al. (2013) | Peer Reviewed Journal | Other: Finland | Other: Population register study | Case control study | Yes | Genetic and Environmental Factors |
| Palmer et al. (2015) | Peer Reviewed Journal | Viet Nam | Unclear | Qualitative research | No | Services and Supports |
| Nguyen et al*.* (2016) | Peer Reviewed Journal | Viet Nam | Unclear | Other: Case study | Yes | Genetic and Environmental Factors |
| Ha (2018) | Other | Viet Nam | Unclear | Other: Case study | Unclear | Infrastructure and Surveillance |
| Tran, Pham, Mai, Le, and Nguyen (2020) | Peer Reviewed Journal | Viet Nam | Unclear | Qualitative research | Unclear | Services and Supports |
| Hsu, Chao, Huang, Bezyak, and Ososkie (2019) | Peer Reviewed Journal | Viet Nam | Other | Cohort study | Unclear | Infrastructure and Surveillance |
| Nguyen et al. (2021) | Peer Reviewed Journal | Viet Nam | Hospital | Cross sectional study | Unclear | Screening and Diagnosis |
| Thuc, Thi, Ngoc, and Vinh (2021a) | Peer Reviewed Journal | Viet Nam | Unclear | Prevalence study | Unclear | Screening and Diagnosis |
| Tran et al. (2020) | Peer Reviewed Journal | Viet Nam | Non-Governmental Organizations (NGO) | Cross sectional study | Yes | Genetic and Environmental Factors |
| Nguyen Thanh et al. (2020) | Peer Reviewed Journal | Viet Nam | Hospital | Other: Uncontrolled clinical trial | Yes | Interventions |
| Thuc, Thi, Ngoc, and Vinh (2021b) | Peer Reviewed Journal | Viet Nam | School | Other: Prevalence | Unclear | Screening and Diagnosis |
| Thuy et al*.* (2020) | Peer Reviewed Journal | Viet Nam | Other: Community | Qualitative research | Unclear | Services and Supports |
| Le and Ha (2021) | Peer Reviewed Journal | Viet Nam | Hospital | Other: Case study | No | Biology |
| Mai and Chaimongko (2022a) | Peer Reviewed Journal | Viet Nam | Hospital | Randomised controlled trial | Yes | Interventions |
| Nguyen (2022) | Peer Reviewed Journal | Viet Nam | Other: Centre for Research and Development of Special Education | Qualitative research | Yes | Interventions |
| Pham et al. (2022) | Peer Reviewed Journal | Viet Nam | Other | Cohort study | No | Genetic and Environmental Factors |
| Pham-The et al. (2022) | Peer Reviewed Journal | Viet Nam | Unclear | Cohort study | No | Genetic and Environmental Factors |
| Mai and Chaimongko (2022b) | Peer Reviewed Journal | Viet Nam | Hospital | Non-randomised experimental study | Yes | Interventions |
| Poon, Cassaniti, Karan, and Ow (2022) | Peer Reviewed Journal | Australia | Tertiary care medical centre; Unclear | Qualitative research | Unclear | Services and Supports |
| Yến-Khanh (2022) | Peer Reviewed Journal | Viet Nam | Other | Qualitative research | No | Infrastructure and Surveillance |
| Yến-Khanh (2023a) | Peer Reviewed Journal | Viet Nam | Other | Qualitative research | Unclear | Infrastructure and Surveillance |
| Yến-Khanh (2023b) | Peer Reviewed Journal | Viet Nam | Other | Qualitative research | No | Infrastructure and Surveillance |
| Vui et al*.* (2023) | Peer Reviewed Journal | Viet Nam | Other | Cross sectional study | Yes | Genetic and Environmental Factors |
| Trinh et al. (2023) | Peer Reviewed Journal | Viet Nam | Hospital | Case control study | Yes | Interventions |
| Nguyen and Tran (2023) | Peer Reviewed Journal | UK | Hospital | Cross sectional study | Unclear | Screening and Diagnosis |
| Smith et al. (2023a) | Peer Reviewed Journal | Australia | Other | Qualitative research | Unclear | Infrastructure and Surveillance |
| Smith et al*.* (2023b) | Peer Reviewed Journal | Australia | Other: Online | Qualitative research | Yes | Services and Supports |
| Nguyen and Nguyen (2023) | Peer Reviewed Journal | Viet Nam | Other: 2 public and 13 private institutions | Cohort study | Unclear | Services and Supports |
| Thao et al. (2023) | Peer Reviewed Journal | Viet Nam | Unclear | Cohort study | No | Genetic and Environmental Factors |
| Le, Rilotta, and Robinson (2024) | Peer Reviewed Journal | Viet Nam | Non-Governmental Organizations (NGO) | Qualitative research | No | Services and Supports |
| Minh, Nguyen, Nguyen, and Do Thi (2024) | Peer Reviewed Journal | Viet Nam | School | Cross sectional study | Yes | Biology |
| Ha, Nguyen, Nguyen, and Tran Thien (2024) | Not Peer Reviewed Journal | Viet Nam | Hospital | Cross sectional study | Yes | Services and Supports |
| Giang, Ha, and Chi (2024) | Peer Reviewed Journal | Viet Nam | School; Other: Home | Cohort study | Unclear | Screening and Diagnosis |
| Bui et al*.* (2024) | Peer Reviewed Journal | Viet Nam | Hospital | Diagnostic test accuracy study | Yes | Genetic and Environmental Factors |
| Arntzen, Halstadtro, Bjerke, and Halstadtro (2010) | Peer Reviewed Journal | Other: Norway | School | Other: Case Study | Unclear | Interventions |
| Ha et al. (2014) | Peer Reviewed Journal | Viet Nam | Hospital ; School; Other: Parent Support Group | Qualitative research | Unclear | Services and Supports |
| Nishijo et al. (2014) | Peer Reviewed Journal | Viet Nam | Hospital | Non-randomised experimental study | No | Genetic and Environmental Factors |
| Tran et al. (2015) | Peer Reviewed Journal | Viet Nam | Other | Cross sectional study | Unclear | Services and Supports |
| Tran et al. (2016) | Peer Reviewed Journal | Viet Nam | Other | Cohort study | No | Genetic and Environmental Factors |
| Ha and Whittaker (2016) | Peer Reviewed Journal | Viet Nam | Other: Community organisation (Hanoi Club of Parents of Children with Autism) | Qualitative research | Unclear | Services and Supports |
| Ha, Whittaker, and Rodger (2017) | Peer Reviewed Journal | Viet Nam | Unclear | Qualitative research | Yes | Infrastructure and Surveillance |
| Fogler, Kuhn, Prock, Radesky, and Gonzalez-Heydrich (2019) | Peer Reviewed Journal | United States | Hospital | Other: Case study | Yes | Screening and Diagnosis |
| Ritter, Terjesen, and Khuc (2019) | Peer Reviewed Journal | Viet Nam | Unclear | Cross sectional study | Unclear | Services and Supports |
| Hoang et al. (2019) | Peer Reviewed Journal | Viet Nam | Other: District Health Center and Center of Population and Family Planning | Cross sectional study | Yes | Screening and Diagnosis |
| Nguyen et al. (2019) | Peer Reviewed Journal | Viet Nam | Tertiary care medical centre | Diagnostic test accuracy study | No | Screening and Diagnosis |
| Lo and Bui (2020) | Peer Reviewed Journal | United States | Other: Homes and community organization | Qualitative research | Unclear | Services and Supports |
| Nguyen, Tran, Thach, and Van Nguyen | Peer Reviewed Journal | Viet Nam | Tertiary care medical centre; Hospital | Other: Not an empirical study | Unclear | Services and Supports |
| Ha and Whittaker (2023) | Peer Reviewed Journal | Viet Nam | Tertiary care medical centre; Non-Governmental Organizations (NGO); Hospital ; School | Qualitative research | Unclear | Services and Supports |
| Cao et al. (2023) | Peer Reviewed Journal | Viet Nam | Other: Online | Cross sectional study | Unclear | Interventions |
| Truong, Mire, Day, Ni, and Keller-Margulis (2022) | Peer Reviewed Journal | Viet Nam | Unclear | Qualitative research | Unclear | Services and Supports |
| Nguyễn and Đỗ (2024) | Peer Reviewed Journal | Viet Nam | School | Cross sectional study | No | Screening and Diagnosis |
| Đào et al. (2024) | Peer Reviewed Journal | Viet Nam | School | Other: case study | No | Interventions |
| Thu Phương (2024) | Peer Reviewed Journal | Viet Nam | School | Other: case study | No | Interventions |
| Thị Thu Thủy (2024) | Peer Reviewed Journal | Viet Nam | Non-Governmental Organizations (NGO) | Cross sectional study | No | Lifespan |
| Thi Anh Phuong, Tuyet Anh, and Thi Thuy (2024) | Peer Reviewed Journal | Viet Nam | School | Cross sectional study | No | Interventions |
| Thị Phương and Thu Giang (2024) | Peer Reviewed Journal | Viet Nam | School | Cross sectional study | No | Services and Supports |
| Phan (2024) | Peer Reviewed Journal | Viet Nam | Non-Governmental Organizations (NGO); School | Cross sectional study | No | Screening and Diagnosis |
| Bá Luyến et al. (2024) | Peer Reviewed Journal | Viet Nam | Non-Governmental Organizations (NGO) | Cross sectional study | Unclear | Lifespan |
| Võ et al. (2024) | Peer Reviewed Journal | Viet Nam | School | Cohort study | No | Services and Supports |
| Nguyễn and Nguyễn (2024) | Peer Reviewed Journal | Viet Nam | School | Cross sectional study | Unclear | Services and Supports |
| Nguyễn and Lê (2024) | Peer Reviewed Journal | Viet Nam | School | Cross sectional study | No | Services and Supports |
| Nguyễn and Đỗ (2025) | Peer Reviewed Journal | Viet Nam | Tertiary care medical centre; School | Cross sectional study | Unclear | Screening and Diagnosis |
| Nguyễn (2024) | Peer Reviewed Journal | Viet Nam | Non-Governmental Organizations (NGO) | Cohort study | No | Lifespan |
| Dân and Giang (2025) | Peer Reviewed Journal | Viet Nam | Unclear | Cross sectional study | No | Services and Supports |
| Phú (2024) | Not Peer Reviewed Journal | Viet Nam | School | Cross sectional study | No | Services and Supports |
| Nguyễn, Phạm, and Phạm (2024) | Peer Reviewed Journal | Viet Nam | Unclear | Cross sectional study | Yes | Interventions |
| Xu et al. (2025) | Peer Reviewed Journal | Viet Nam | Other: Secondary data (GBD) | Prevalence study | Yes | Infrastructure and Surveillance |
| Khanh et al. (2024) | Peer Reviewed Journal | Viet Nam | Hospital | Cross sectional study | Yes | Services and Supports |
| Bui et al. (2024) | Peer Reviewed Journal | Viet Nam | Hospital | Case control study | Yes | Genetic and Environmental Factors |
| Tran (2024) | Peer Reviewed Journal | Viet Nam | School | Prevalence study | Yes | Infrastructure and Surveillance |
| Nguyen-Martinez et al. (2025) | Other | United States | Unclear | Other: Case study | Yes | Screening and Diagnosis |
| Szücs et al. (2025) | Peer Reviewed Journal | Viet Nam | Other: Secondary data | Prevalence study | Yes | Infrastructure and Surveillance |
| Khanh, Mai, and Duong (2025a) | Peer Reviewed Journal | Viet Nam | Hospital | Cross sectional study | Yes | Services and Supports |
| Khanh et al. (2025b) | Peer Reviewed Journal | Viet Nam | Hospital | Cross sectional study | Yes | Services and Supports |
| Dung (2020) | Peer Reviewed Journal | Viet Nam | Unclear | Qualitative research | Unclear | Services and Supports |
| Dieu (2022) | Dissertation | Viet Nam | Other: Online | Cross sectional study | Unclear | Services and Supports |

**Figure 1.**

Count of Studies with Grouped Sample Sizes.

**Figure 2.**

Count of Diagnostic Instruments and Screening Tools Mentioned in Diagnosis Process.

Note. Childhood Autism Rating Scale (CARS); Diagnostic and Statistical Manual of Mental Disorders (DSM); Client Development Evaluation Report (CDER); International Classification of Diseases (ICD); Ages & Stages Questionnaire (ASQ); Developmental Behaviour Checklist Parent/Carer Version (DBC-P); Modified Checklist for Autism in Toddlers (M-CHAT); Autism Diagnostic Observation Schedule (ADOS); Autism Diagnostic Interview-Revised (ADI-R).

**Figure 3.**

Count of Study Settings.

**Figure 4.**

Count of MMAT Criteria.
